# Body fat compartment determination by encoder–decoder convolutional neural network: application to amyotrophic lateral sclerosis

**DOI:** 10.1038/s41598-022-09518-w

**Published:** 2022-04-01

**Authors:** Ina Vernikouskaya, Hans-Peter Müller, Dominik Felbel, Francesco Roselli, Albert C. Ludolph, Jan Kassubek, Volker Rasche

**Affiliations:** 1 Department of Internal Medicine II, Ulm University Medical Center, Ulm, Germany; 2 Department of Neurology, University of Ulm, Ulm, Germany; 3 German Center for Neurodegenerative Diseases (DZNE), Ulm, Germany; 4 Core Facility Small Animal MRI, University of Ulm, Ulm, Germany

**Keywords:** Amyotrophic lateral sclerosis, Computer science

## Abstract

The objective of this study was to automate the discrimination and quantification of human abdominal body fat compartments into subcutaneous adipose tissue (SAT) and visceral adipose tissue (VAT) from T1-weighted MRI using encoder-decoder convolutional neural networks (CNN) and to apply the algorithm to a diseased patient sample, i.e., patients with amyotrophic lateral sclerosis (ALS). One-hundred-and-fifty-five participants (74 patients with ALS and 81 healthy controls) were split in training (50%), validation (6%), and test (44%) data. SAT and VAT volumes were determined by a novel automated CNN-based algorithm of U-Net like architecture in comparison with an established protocol with semi-automatic assessment as the reference. The dice coefficients between the CNN predicted masks and the reference segmentation were 0.87 ± 0.04 for SAT and 0.64 ± 0.17 for VAT in the control group and 0.87 ± 0.08 for SAT and 0.68 ± 0.15 for VAT in the ALS group. The significantly increased VAT/SAT ratio in the ALS group in comparison to controls confirmed the previous results. In summary, the CNN approach using CNN of U-Net architecture for automated segmentation of abdominal adipose tissue substantially facilitates data processing and offers the opportunity to automatically discriminate abdominal SAT and VAT compartments. Within the research field of neurodegenerative disorders with body composition alterations like ALS, the unbiased analysis of body fat components might pave the way for these parameters as a potential biological marker or a secondary read-out for clinical trials.

## Introduction

Accurate segmentation of human body fat compartments from MRI is a challenging task when performed manually, due to the limited reproducibility of manual or semi-manual delineations. Moreover, MRI scans are often not free of magnetic field inhomogeneities and chemical shift artifacts, requiring highly trained experts for segmentation. Semi-automatic segmentations using thresholding and histogram-based region growing have been successfully used to segment the fat compartments with high-contrast images^1,2^, but these methods are limited by high computation costs and long performance times.

Multiple automated and semi-automated segmentation algorithms have been introduced over the past two decades^3–6^. More recently, learning-based methodologies outperforming traditional methods have been proposed to automate the segmentation tasks^7–10^. Concerning organ segmentation, artificial intelligence (AI) and especially convolutional neural networks (CNNs) have proven to be able to model all variations found on a training dataset and to rapidly perform an automatic segmentation without high computational requirement^11^. CNN are biologically inspired machine learning concepts which are used in machine processing of image data^12^. The nomenclature is deducted from neural networks in the human (or animal) brain, illustrating a self-learning concept to identify e.g. structural characteristics in images. Methods based on fully convolutional networks of encoder-decoder architectures are promising approaches to automated segmentation of adipose tissue on abdominal water-fat Dixon MRI^13,14^.

Amyotrophic lateral sclerosis (ALS) is the most frequent adult onset neurodegenerative motor neuron disease with a prevalence of 2.6–3.0 cases per 100,000 people in European populations and is characterized by the progressive degeneration of both upper motor neurons and lower motor neurons, leading to death through respiratory insufficiency in most patients within about three years after disease onset^15^. In ALS, riluzole prolongs lifespan for a few months and individual treatment options have to be considered for improving survival, symptom control, and social participation—however, ALS is not curable^16^. Epidemiological data suggest that ALS patients suffer from catabolism^17^ and begin to lose weight more than 10 years before the onset of motor symptoms^18^. Catabolism may result from the combination of dysphagia and intrinsic hypermetabolism, as shown in ALS patients as well as a mouse model of ALS^19^. In the disease course of ALS, weight loss and metabolic status are strong predictors of survival^17^.

The search for reliable biomarkers is a high priority in ALS research. In a previous study, it could be demonstrated that metabolic status indirectly assessed by body fat distribution is a predictor of survival in ALS^20^. Given that a recent clinical trial demonstrated a survival benefit by high-caloric fatty diet for fast-progressing ALS patients^19^, a non-invasive biomarker assessment of the body composition in ALS gains additional clinical relevance. As a marker, ALS patients have been shown to display an expanded ratio between visceral (VAT) and subcutaneous adipose tissue (SAT), indicating that adipose tissue is also affected in its topography and potentially its function in ALS^20^. Most remarkably, a similar although less pronounced pattern was observed in pilot studies in other neurodegenerative diseases with existent but much less obvious weight loss in their advanced disease stages like Parkinson´s disease^21^ and, as a trend, Alzheimer´s disease^22^.

In the current study, a novel approach is presented that uses a fully convolutional network of traditional U-Net architecture for automated segmentation and separation of SAT and VAT on T1-weighted MR images with a wide range of anatomical variations across subjects and was, as a proof of concept, applied to a cohort of whole body MRI data sets from ALS patients and controls.

## Results

### Performance of neural network classification

**Figure 1:**
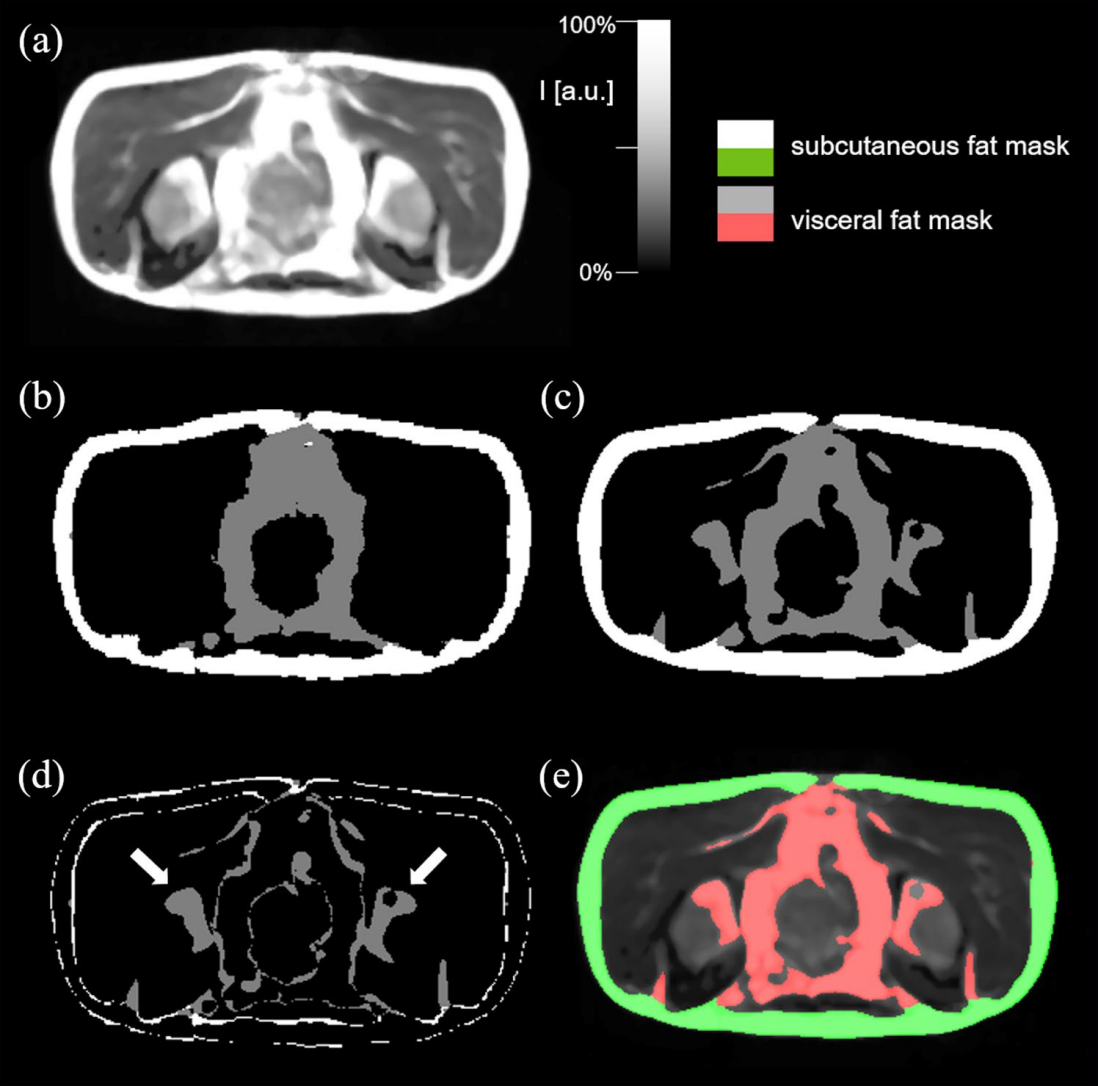
Prediction results from a test dataset from a control. (**a**) Original transversal MRI single plane image. (**b**) Reference segmentation mask. (**c**) Predicted label map. (**d**) Difference image between reference and predicted segmentation with arrows indicating major differences in prediction of VAT in hip bones. (**e**) Predicted segmented mask overlaid on original MR image.

For both validation and test data, the average performance metrics are summarized in Table 1. The neural network was able to generate the segmentations at an average pixel error of 2% for SAT and 4% for VAT, respectively, in the ALS group of the validation set, while an average error below 1% for both fat compartments was achieved in the control group of the validation set. Similar performance was achieved on the test data with an average error below 3.5% in both groups. Dice similarity coefficients in the test data achieved 0.87 for SAT and 0.68 for the VAT compartment. Figure 1 shows the comparison of the segmentation performed on a single plane MR image from a control test dataset (Fig. 1a) by the reference method (Fig. 1b) and CNN (Fig. 1c), in addition visualized as a difference image (Fig. 1d); differences appear in bones and at the edges of structures suggesting that in comparison to the reference technique the neural networks tend to a smoothed segmentation without pixelated edges. The overlay of predicted SAT and VAT compartments on the original MR image is shown in Fig. 1e.

**Table 1:** Average network performance in validation and test data sets. VAT—visceral adipose tissue; SAT—subcutaneous adipose tissue.

	Fat compartment	Metric	Validation	Test
ALS patients	VAT	Dice	0.56 ± 0.09	0.68 ± 0.15
		Pixel error (%)	4.11 ± 1.31	3.11 ± 1.29
	SAT	Dice	0.87 ± 0.06	0.87 ± 0.08
		Pixel error (%)	2.07 ± 0.80	1.55 ± 0.63
Controls	VAT	Dice	0.60 ± 0.10	0.64 ± 0.17
		Pixel error (%)	0.79 ± 0.48	2.41 ± 1.67
	SAT	Dice	0.87 ± 0.03	0.87 ± 0.04
		Pixel error (%)	0.69 ± 0.08	2.11 ± 1.37

**Figure 2:**
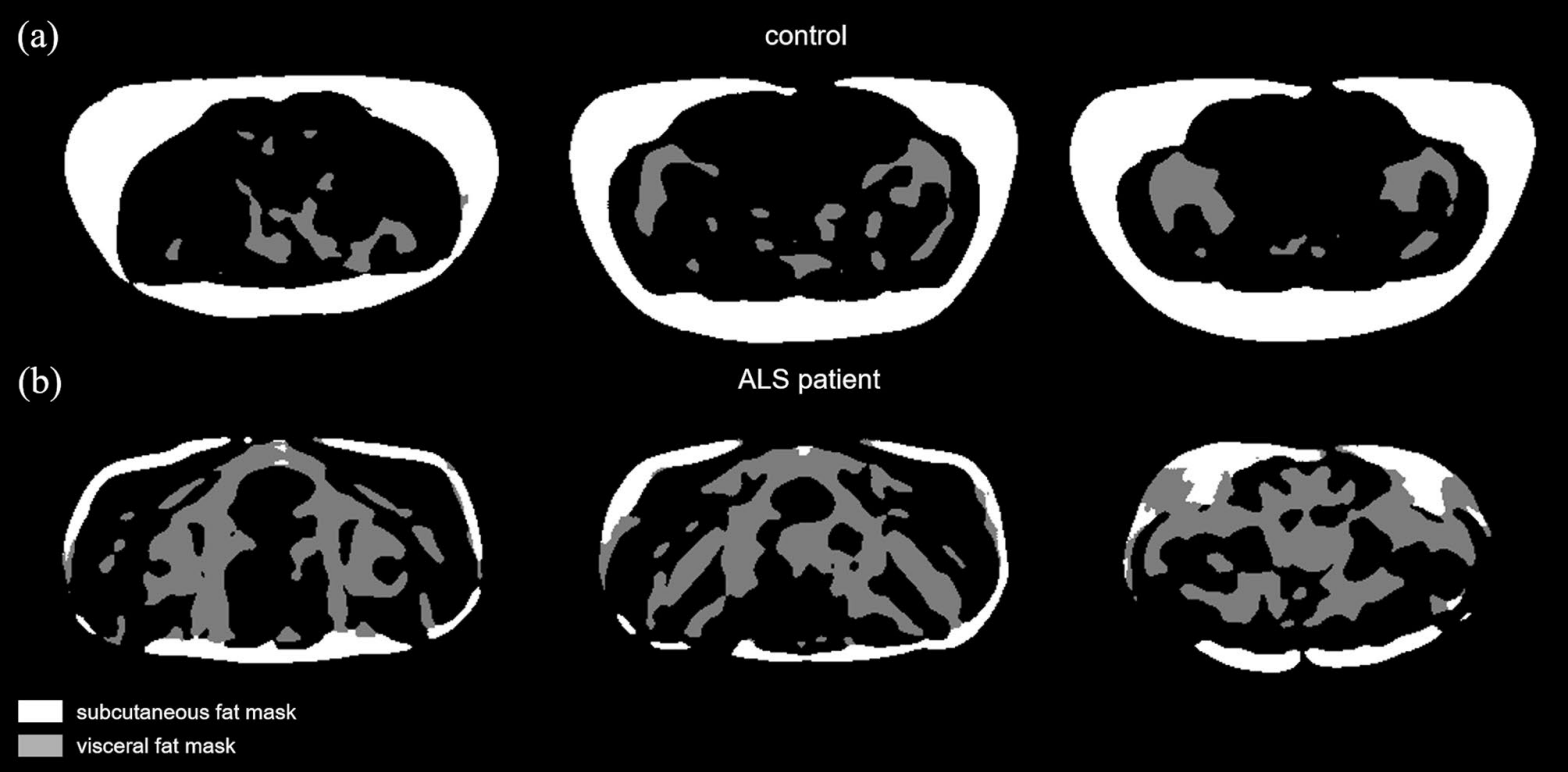
Prediction results on randomly selected test cases. Multi-slice prediction on a dataset from a control (**a**) and from an ALS patient (**b**).

**Figure 3:**
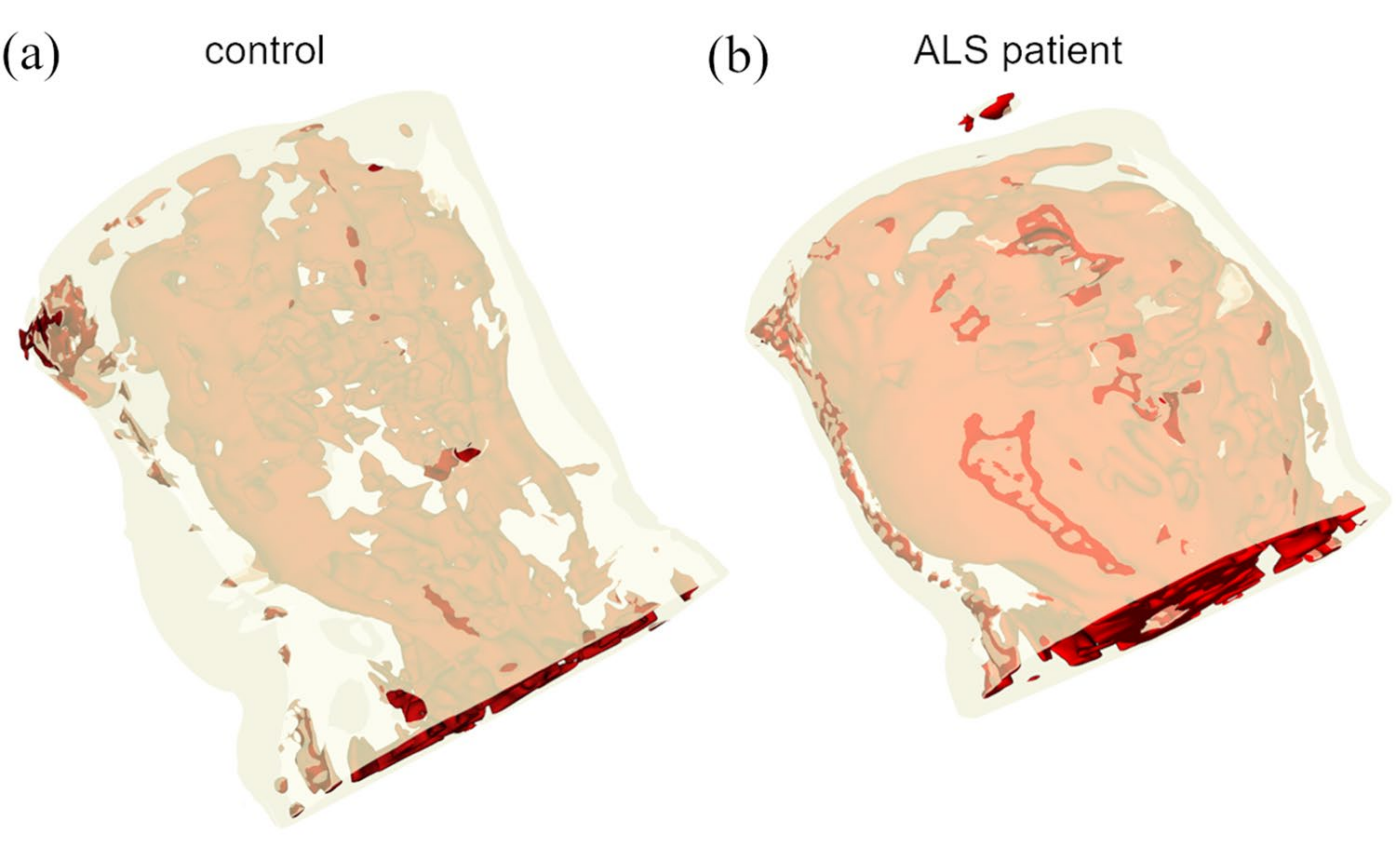
3D rendering of multi-slice predictions from a control (**a**) and ALS patient (**b**).

The predicted segmentation of single slices for two test datasets from a control (Fig. 2a) and from an ALS patient (Fig. 2b) exemplarily demonstrates the difference in distribution of the VAT and SAT compartments. The 3D models rendered from multi-slice predictions of control and ALS patient are shown in Fig. 3.

### Correlation of CNN classification results and reference classification results

**Figure 4:**
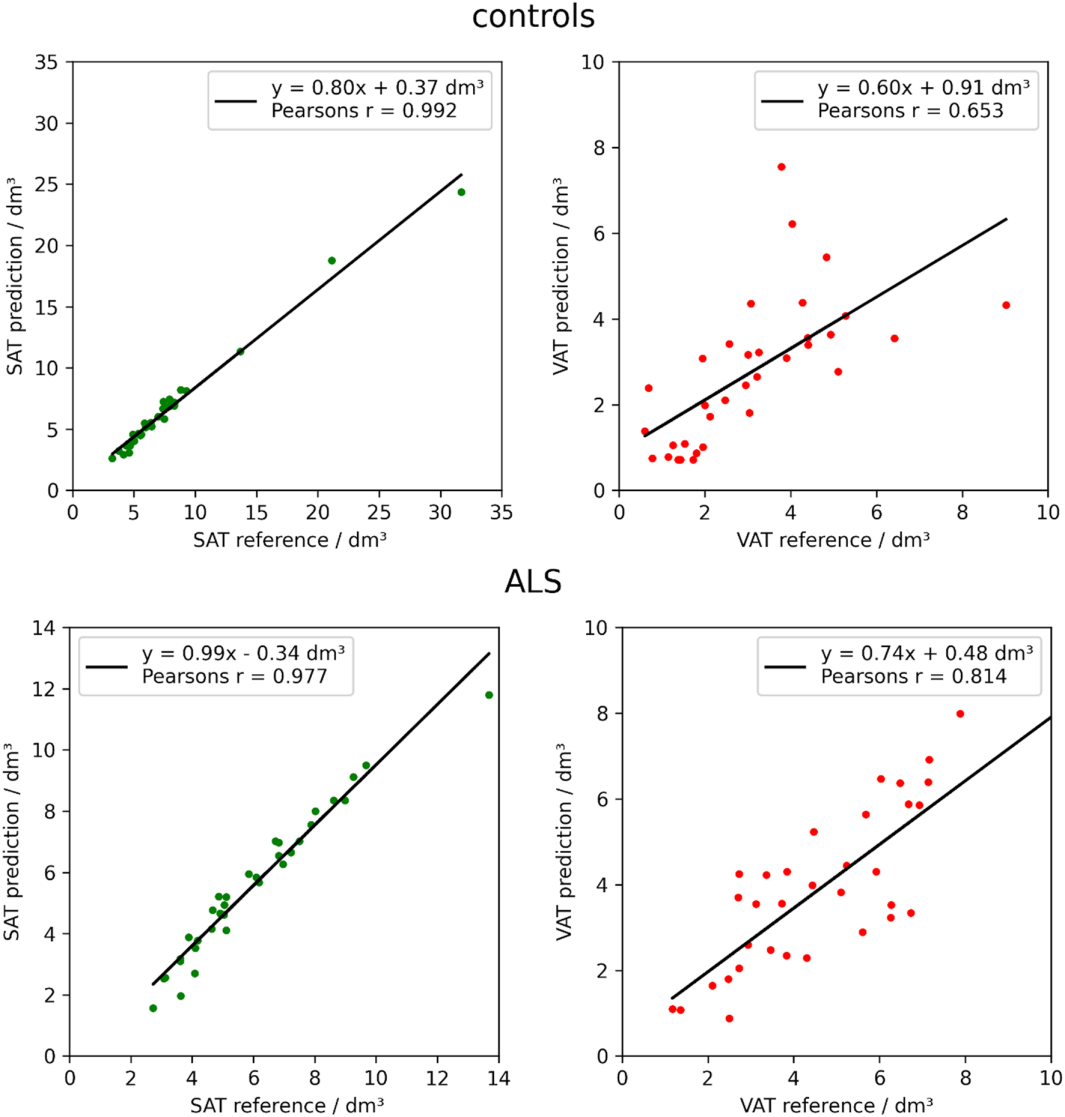
Correlations between volumes of subcutaneous adipose tissue (SAT) and visceral adipose tissue (VAT) calculated based on the reference segmentation vs. predicted by U-Net in the control group (upper panels) and in the ALS group (lower panels).

Figure 4 shows the correlation between the SAT and VAT volumes calculated from the reference segmentation and predicted segmentation on the test data from control and ALS groups. A significant linear correlation with Pearson coefficients *r* = 0.992 in controls and *r* = 0.977 in ALS patients was observed for SAT, while lower Pearson coefficients *r* = 0.653 in controls and *r* = 0.814 in ALS patients were obtained for VAT. The most commonly observed errors in segmentation with the U-Net were erroneous interpretation of the hip bones as VAT and errors in discrimination between VAT and SAT in ambiguous areas (Fig. 2).

### Application of body fat compartment ratio assessment to ALS

**Figure 5:**
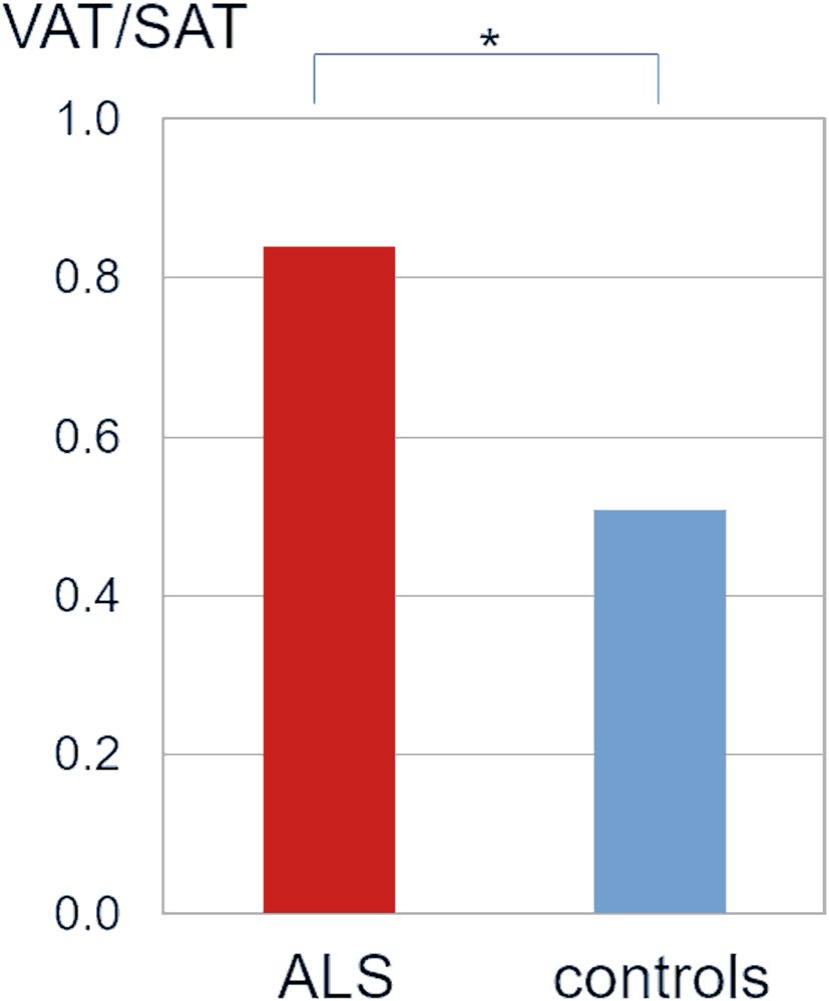
Ratio of visceral adipose tissue (VAT) and subcutaneous adipose tissue (SAT) for 34 ALS patients vs. 34 controls (test sample data). Error bars are the standard error of the mean; *p < 0.01.

The analysis at the group level revealed a significant difference for the body fat compartment ratio when comparing ALS patients versus controls. For the test data sample (34 ALS patients, 34 controls), VAT/SAT ratio was significantly increased in the ALS patient group (*p* = 0.002) (Fig. 5). No significant correlations of VAT/SAT ratio with disease duration, ALS Functional rating scale-revised (ALS-FRS-R) score, or ALS-FRS-R slope, respectively, could be detected. Also, no correlation between VAT and SAT volumes for both groups could be found.

## Discussion

In this study, a deep learning pipeline was established, validated, and implemented to differentiate and quantify the components of abdominal adipose tissue. It was possible to automatically generate robust VAT and SAT label maps using fully convolutional network of U-Net architecture on the given T1-weighted whole-body MRI data with a wide range of anatomical variation, i.e. body fat composition, across control and patient groups. Pairwise comparison of the predicted label maps with the reference segmentation yielded the major disagreement in the segmentation of VAT around the spine and hip bones and hyperintense regions in SAT.

There are previously published advanced data processing pipelines to segment and quantify adipose tissue based on deep learning methods. One of these methods, i.e., FatSegNet with two 2-D competitive dense fully convolutional networks, was successfully applied to the abdominal Dixon MR images acquired in a large population‐based cohort of healthy subjects in the Rhineland study with high reliability^14^, but, to the best of our knowledge, has not been applied to patient data yet.

### Limitations

The potential for further optimization is dependent on the quality of the reference segmentation, which on its turn is limited by factors like level set-based nature of the method, image contrast, and (breathing-related) motion artifacts. Especially when patients with a high disease burden (like ALS patients) undergo MRI, the data quality is sometimes limited due to motion or further influencing factors. Given that these data are much more prone to contain limited data quality than data from healthy volunteers, the current application of the technical solution to whole body MRI acquired in severely affected patients constitutes a strength of the approach over those techniques which have been applied solely to healthy subjects.

### Summary

The current results in the T1w MRI data in the ALS patient cohort could reproduce the results of a previous study with a semi-automatic technique^20^. It seems safe to conclude that the CNN technique could act as a valuable and robust operator-independent tool for future studies that investigate body fat compartments by structural whole body MRI in a cross-sectional and potentially in a longitudinal design. The unbiased approach on the one hand and the proof of concept in the ALS patients´ data on the other hand suggest its use in natural history registries^23^ or clinical trials^24^ which address changes in the body composition and associated potential benefit in clinical parameters or prognosis in neurodegenerative disorders like motor neuron diseases. CNN-based quantification of VAT/SAT might serve as a potential biological marker or a secondary read-out to monitor specific body composition changes which are more specific than the clinical parameter BMI in order to improve our understanding of the metabolic pathology.

## Subjects and methods

### Subjects

One-hundred-and-fifty-five participants underwent T1-weighted examination on a 1.5 T MRI scanner; 81 healthy subjects composed the control group, whereas 74 ALS patients composed the ALS group. Seventy-four ALS patients were recruited in the outpatient and inpatient settings of the Department of Neurology, University of Ulm, Germany. The age- and gender-matched control group consisted of 81 healthy subjects who had been recruited through a volunteer panel (University for the Aged, volunteer work exhibition) or spouses of patients. Exclusion criteria for controls were any neurological/psychiatric disease or other medical condition or general contraindications to acquire MRI. Exclusion criteria for ALS patients were also general contraindications for MRI acquisition, in addition reduced respiratory function (FVC < 40%).

All patients were diagnosed with definite or (clinically or laboratory-supported) probable ALS using the revised version of the El Escorial World Federation of Neurology criteria^25^ and had a disease duration of 25 ± 19 months (with disease onset defined as date of first muscle weakness, excluding fasciculation and cramps), an average ALS-FRS-R score of 37 ± 8, and an ALS-FRS-R slope of − 10 ± 10 per year. All patients received neurophysiological studies during the clinical diagnostic process. Fifty ALS patients had a spinal onset and 14 had a bulbar onset; 3 ALS patients had a documented frontal involvement, and forced vital capacity (FVC) was available in 10 patients with an average value of 65% ± 14%. The patients underwent a neurological interview to investigate the age, disease duration and the presence of bulbar symptoms. The information on diabetes mellitus and dyslipidemia were based on patient history, no laboratory data for these conditions were acquired in the study. Height and weight were measured in order to calculate the BMI. The controls did not take any medication; 63 (85%) of the ALS patients took riluzole. Details are summarized in Table 2. The study was performed in accordance with the ethical standards of the latest revision of the Declaration of Helsinki and was approved by the ethical review committee of Ulm University (reference 179/2008). A written informed consent was obtained from all participants.

**Table 2:** Clinical characterization of subject groups. FVC—forced vital capacity.

	ALS patients	controls	*p*
m/f	50/24	42/39	0.05
Age/years	60 ± 13 median 62 range (22–81)	60 ± 13 median 58 range (26–88)	0.19
BMI/kg/m^2^	26 ± 4 median 24 range (15–32)	24 ± 4 median 25 range (19–40)	0.01
ALS-FRS-R	37 ± 8	–	–
Slope (ALS-FRS-R)/year	− 10 ± 10	–	–
Disease duration/years	2.1 ± 1.6	–	–
Onset (spinal/bulbar)	50/14	–	–
FVC/%	65 ± 14	–	–
Age at onset/years	58 ± 12	–	–

### MRI protocol

MRI data were acquired on a 1.5 T scanner (Symphony, Siemens Medical, Erlangen, Germany). The whole body MRI scans were recorded by acquisition of 6 to 8 3-D volumes with a standard T1 weighted spin-echo sequence, each consisting of 36 2-D slices with a slice thickness of 6 mm and an in-plane resolution of 1.25 mm × 1.25 mm. Repetition time was 476 ms and time to echo was 12 ms. The total acquisition time for one volume was 4:30 min. Slices were recorded with no gap. To confirm that no gap is left in between the consecutive volumes, an overlap of about 6 to 18 mm was chosen between the volumes so that a total area of about 120 cm was scanned^26^.

### Data preprocessing

Data preprocessing was performed by the pipeline already performed and documented in a previous study^18^ and was performed by the in-house developed software package *ATLAS* (Automatic Tissue Labelling Analysis Software)^26^. The preprocessing consisted of several steps, detailed in the following.

Each of the single recorded 3-D volumes covered a body section of about 22 cm. In between two consecutively recorded 3-D-volumes, an overlap of between 6 and 18 mm, i.e. 1 to 3 slices, allowed for the application of a conjugated simplex fitting method with 3 degrees of freedom (x, y, z—translational shift). That way, one contiguous body volume was merged. Prior to subsequent analysis, the data were supersampled to isotropic voxels with size 1.2 × 1.2 × 1.2 mm^3^. In cases that magnetic field or gradient inhomogeneities or distortions might cause image inhomogeneities over the large scanning area, an interactive repair functionality was used in order to correct the distortions and prepare the data set for further data operations. As the arms where often only partially recorded and were not of interest for further analysis, the arms were manually deleted from the data sets. Finally, in order to homogenize intensity in the data sets, median filtering was applied.

### Reference method

Subcutaneous fat determination was performed using the ARTIS algorithm (Adapted Rendering for Tissue Intensity Segmentation) which has already proven to reveal high stability in the results; abdominal visceral fat tissue was identified by selecting all connected voxels with respect to their intensity within the from ARTIS predefined range^26^.

### CNN-based method

All available data were split in training (50%), validation (6%), and test (44%) data, based on age and BMI strata to ensure a balanced population distribution. In total 22,269 slice images (11,485 from the control group and 10,784 from the ALS group) of 384 × 384 pixels resolution were used for the training process, in which the learnable parameters of the network were adjusted. In total, 2483 images (1651 from the control group and 832 from the ALS group) were used for validation phase, in which a chosen network configuration together with its learned parameters was evaluated. Successful network configuration was then tested on 9765 images from the control group and 10,036 images from the ALS group. For each single transversal slice in the MRI examination, an image sample consisting of a lossless grayscale image (Fig. 1a) and corresponding mask (Fig. 1b) segmented using reference method was created. The segmented mask contains three classes of pixels including the SAT (pixel values of 255), the VAT (pixel values of 127), and the background (pixel values of 0).

**Figure 6:**
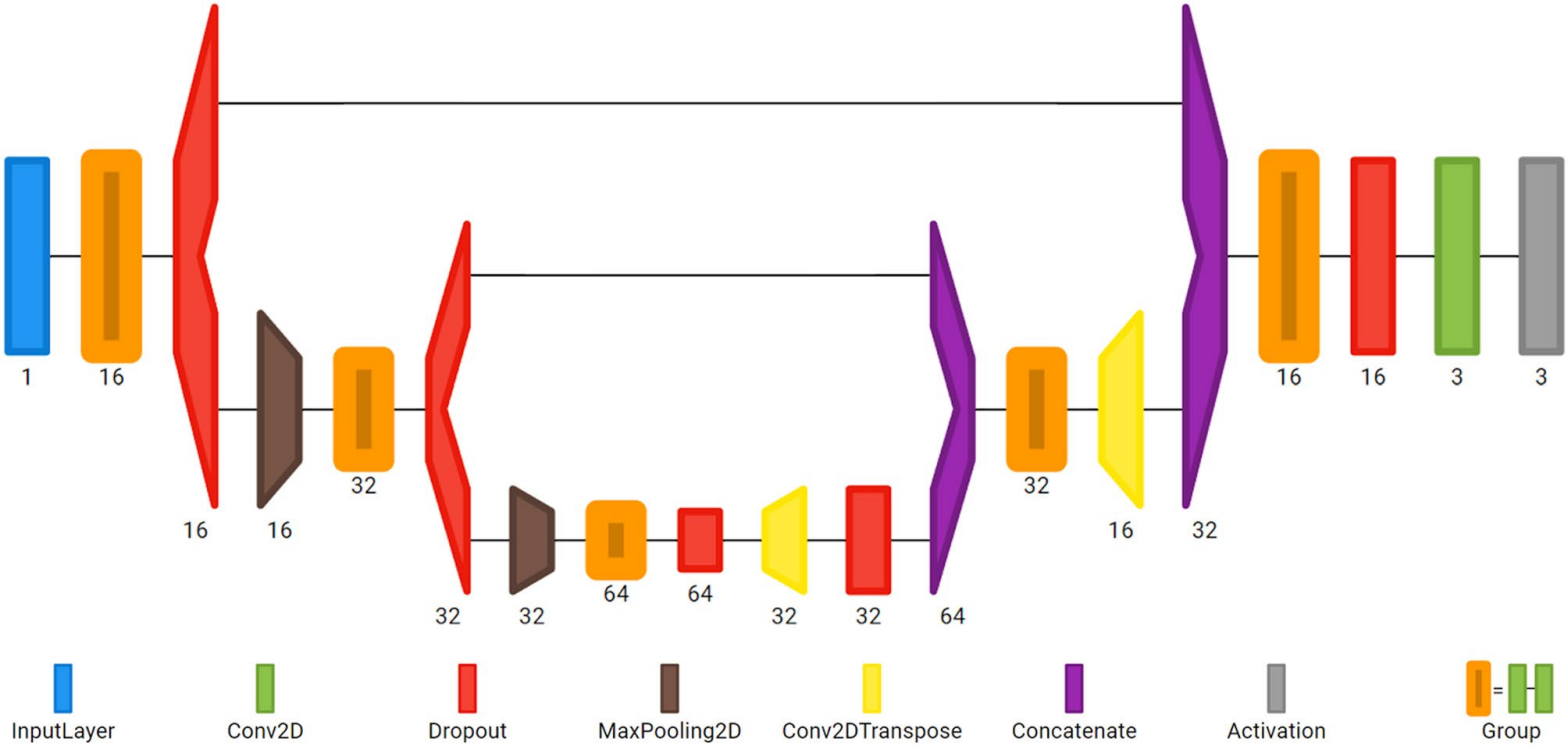
Architecture of proposed encoder-decoder convolutional neural network. It takes an image of 384 × 384 pixels resolution and process it in several convolutional, pooling, transposed convolutional and concatenation layers, before the final pixelwise semantic segmentation is performed with the “softmax” activation in the last classification layer.

The proposed method is based on an encoder-decoder U-net architecture, consisting of a convolutional part downsampled with the maxpool layer and strided transposed convolutional upsampling part in combination with drop out regularization. The architecture of the proposed network visualized with Net2Vis tool^27^ is shown in Fig. 6. In contrast to original architecture, three down-sampling steps were used and the number of feature channels in the contracting path was reduced to 16, 32, and 64, respectively. The model was implemented in Keras and trained on the introduced training dataset on a GeForce GTX 1060 6 GB GPU for 15 epochs with the batch size of 16 samples per pass with the adaptive moment estimation algorithm. Categorical cross-entropy was used as a loss function for multi-class semantic segmentation.

### Performance evaluation and fat quantification

The segmentation performance of the network was evaluated in terms of average dice similarity coefficients calculated through all slices in all examinations in both validation and test datasets as spatial overlap between the predicted (P) and reference (R) segmentation for a given class. The dice score index is defined as:

1 $$ dice = \frac{{2 \cdot \left| {RP} \right|}}{\left| R \right| + \left| P \right|}, $$

where $\left| R \right|$ and $\left| P \right|$ represent the number of elements in each label map and $\left| {RP} \right|$ the number of intersecting elements.

Further average pixel error in percentage was calculated through all slices for all examinations as number of pixels which are not intersecting in predicted and reference label maps in relation to image size in pixels:

2 $$ E = \left( {1 - \frac{{\left| {RP} \right|}}{W \cdot H}} \right), $$

where *E* denotes the error, *W* and *H* denote the image width and height.

Pixelwise computation of segmented SAT and VAT through all slices of a single test object was performed for volumetric quantification of the corresponding fat compartments with both methods.

### Statistical analysis

Statistical analysis was performed using Microsoft^®^ Excel^®^ 2019 MSO and Python library SciPy version 1.6.1 including statistical analysis module. Continuous data were presented as mean ± standard deviation (SD) or median (range) and categorical numbers with percentages. Comparisons between two groups were conducted using a two-tailed Student’s t-test. A two-tailed *p*-value of less than 0.05 was considered statistically significant. Associations to clinical parameters disease duration, ALSFRS-R score, and disease onset were calculated by Pearson correlation.

### Ethical approval

All human studies have been approved by the appropriate ethics committee and have therefore been performed in accordance with the ethical standards laid down in the 1964 Declaration of Helsinki and its later amendments. The ethical review committee of the University of Ulm approved this study (# 179/2008).

### Informed consent

Informed consent was obtained from all individual participants included in the study.

## Data Availability

The original contributions presented in the study are included in the article, further inquiries can be directed to the corresponding author/s.
